# Risk of depression in pregnancy among pregnant women undergoing high-risk prenatal care

**DOI:** 10.1590/1980-220X-REEUSP-2021-0470en

**Published:** 2022-07-18

**Authors:** Gabriela de Magalhães Ribeiro, Julia Ferreira Cieto, Mônica Maria de Jesus Silva

**Affiliations:** 1 Universidade de São Paulo, Escola de Enfermagem de Ribeirão Preto, Ribeirão Preto, SP, Brazil.

**Keywords:** Depression, Pregnancy, Prenatal Care, Risk Factors, Obstetric Nursing, Depresión, el Embarazo, Atención Prenatal, Factores de Riesgo, Enfermería Obstétrica, Depressão, Gravidez, Cuidado Pré-Natal, Fatores de Risco, Enfermagem Obstétrica

## Abstract

**Objective:**

to identify the risk of depression in pregnancy among pregnant women undergoing follow-up in high-risk prenatal care, to assess the factors associated with higher risk of depression in pregnancy and to compare the risk of depression in each gestational trimester.

**Method:**

this is a descriptive, correlational, cross-sectional study, conducted with 151 pregnant women in prenatal care in a high-risk pregnancy outpatient clinic at a university hospital in the state of São Paulo, Brazil. Data were collected through an online form. Chi-square and Fisher’s exact tests were performed. After the bivariate analysis, the variables were included in the logistic regression model. In the final model, the Odds Ratio was calculated.

**Results:**

118 (78.1%) pregnant women had a higher risk of depression during pregnancy, which was higher in the first trimester, but without statistical significance. The number of pregnancies (OR = 0.32) and marital status (OR = 0.07) remained significantly associated with higher risk of depression during pregnancy as protective factors.

**Conclusion:**

the results elucidate the importance of screening for depression risk and the significant need to improve access to effective interventions for preventing prenatal depression and promoting mental health.

## RESUMO

**Objetivo:**

identificar o risco de depressão na gravidez entre gestantes em acompanhamento na assistência pré-natal de alto risco, avaliar os fatores associados ao maior risco de depressão na gravidez e comparar o risco de depressão em cada trimestre gestacional.

**Método:**

estudo descritivo, correlacional, transversal, realizado com 151 gestantes em acompanhamento pré-natal em ambulatório de gestação de alto risco de um hospital universitário no estado de São Paulo, Brasil. Os dados foram coletados por um formulário *online*. Foram realizados os Testes do Qui-Quadrado e Exato de Fisher. Após a análise bivariada, as variáveis foram incluídas no modelo de regressão logística. No modelo final, foi calcula a Razão de Chances.

**Resultados:**

118 (78,1%) gestantes apresentaram maior risco de depressão na gravidez, o qual foi maior no primeiro trimestre, mas sem significância estatística. O número de gestações (OR = 0,32) e o estado civil (OR = 0,07) permaneceram associados significativamente ao maior risco de depressão na gravidez como fatores de proteção.

**Conclusão:**

os resultados elucidam a importância do rastreamento do risco de depressão e a necessidade significativa de melhorar o acesso a intervenções eficazes para prevenção da depressão pré-natal e promoção da sua saúde mental.

## RESUMEN

**Objetivo:**

identificar el riesgo de depresión en el embarazo entre gestantes en control prenatal de alto riesgo, evaluar los factores asociados a mayor riesgo de depresión en el embarazo y comparar el riesgo de depresión en cada trimestre gestacional.

**Método:**

estudio descriptivo, correlacional, transversal, realizado con 151 gestantes en control prenatal en un ambulatorio de embarazo de alto riesgo de un hospital universitario del estado de São Paulo, Brasil. Los datos se recopilaron mediante un formulario en línea. Se realizaron las Pruebas Chi-Cuadrado y Exacta de Fisher. Después del análisis bivariado, las variables se incluyeron en el modelo de regresión logística. En el modelo final se calculó el Odds Ratio.

**Resultados:**

118 (78,1%) gestantes presentaron mayor riesgo de depresión durante el embarazo, siendo mayor en el primer trimestre, pero sin significación estadística. El número de embarazos (OR = 0,32) y el estado civil (OR = 0,07) permanecieron significativamente asociados con un mayor riesgo de depresión en el embarazo como factores protectores.

**Conclusión:**

los resultados aclaran la importancia de la detección del riesgo de depresión y la necesidad significativa de mejorar el acceso a intervenciones efectivas para prevenir la depresión prenatal y promover la salud mental.

## INTRODUCTION

Pregnancy is a time of vulnerability for the onset or relapse of mental illness^(1)^. In this period, depression is the most prevalent psychiatric disorder, with symptoms ranging from mild to severe^(2,3)^. A systematic review of studies conducted in developed and low-income countries reported a prevalence of prenatal depression in the range of 15.6% to 31.1%^(1)^.

The occurrence of major depressive disorder, referred to in this article as depression, requires the presence of five or more symptoms over a two-week period. At least one of the symptoms reported must be depressed mood or loss of interest or pleasure in usual activities of daily living. The other symptoms of depression, according to the Diagnostic and Statistical Manual of Mental Disorders (DSM-V), are depressed mood most of the day, sadness, loss of interest or pleasure in all or almost all activities, loss or gain of significant weight, insomnia or hypersomnia, psychomotor agitation or retardation, fatigue or loss of energy, feelings of worthlessness or excessive and/or inadequate guilt, indecision or diminished ability to think or concentrate, and recurrent thoughts of death^(4)^.

When it occurs during pregnancy, depression is called prenatal^(4)^, and is associated with adverse maternal, child and family well-being^(5)^. When untreated, depression in pregnancy results in negative neonatal outcomes^(6)^, adverse obstetric outcomes^(7)^, negative social and personal adjustments in the mother^(8,9)^, and prediction for postpartum depression^(10)^.

In pregnancy, some women are at higher risk of developing depression, while others remain resilient, even in the face of adversity^(1)^. In women with clinically defined obstetric complications that result in additional risk of pregnancy, defined as high-risk pregnancies, there is an even greater possibility of serious psychological problems and, particularly, depression in pregnancy^(11)^.

In this context, identifying the risk of depression in pregnancy, not the occurrence of the disorder, is healthy, when tracing a scenario of a pregnant woman’s vulnerability, preventing the occurrence of the disorder and interrupting the illness process early.

To this end, the administration of an instrument to identify women at risk of depression during pregnancy should be a universal practice in order to promote the long-term well-being of women and their children^(1)^.

In this context, a specific instrument was developed and validated in Brazil to assess the risk of depression during pregnancy, which considers the specificities of pregnancy and the Brazilian sociocultural characteristics^(12)^.

Knowledge of the risk of depression in pregnancy allows for the development of early interventions for monitoring and promoting women’s mental health during pregnancy, timely referral to specialized mental health care, and cost reduction for the health system.

The present study addresses this relevant issue and invests efforts in detecting the risk of depression in pregnancy among pregnant women undergoing high-risk prenatal care, considering that, in Brazil, there are few studies in this follow-up. Thus, it is urgent to identify the risk of depression in pregnancy, in order to support actions to promote pregnant women’s mental health.

Thus, this study aimed to identify the risk of depression in pregnancy among pregnant women in high-risk prenatal care, to evaluate the possible factors associated with a higher risk of depression in pregnancy and to compare the risk of depression in each gestational trimester.

## METHOD

### Design of Study

This is a descriptive and correlational cross-sectional study conducted in the city of Ribeirão Preto, São Paulo, Brazil.

### Population

The study population consisted of pregnant women undergoing prenatal care in a high-risk pregnancy outpatient clinic.

### Local

The study was conducted in a high-risk pregnancy outpatient clinic at a university hospital located in the countryside of the state of São Paulo, Brazil.

### Selection Criteria

Pregnant women in prenatal care at a high-risk pregnancy outpatient clinic, aged equal to or greater than 18 years, and at any gestational age were included. Pregnant women that were illiterate were excluded.

### Sample Definition

The number of participants in the sample was calculated according to the number of pregnant women who underwent prenatal care at the high-risk pregnancy outpatient clinic in 2019, according to data from the hospital’s Department of Health Care. For the sample calculation, the parameters considered were prevalence of 50%, confidence level of 95%, margin of error of 5%, composing a sample size of 151 pregnant women.

The prevalence taken as the basis for the sample calculation was assumed to be unknown, given the scarcity of studies that address the risk of depression in pregnancy in the context of high-risk prenatal care. Thus, the recommendation recommended by the literature was followed, which, in order to obtain a conservative estimate of sample size, suggests a prevalence value of 50%, which results in a sample size that includes any p-value^(13)^.

### Data Collection

Data collection was carried out online, after approval by the Research Ethics Committee, from April 28 to August 16, 2021. To do so, the researchers recruited participants by e-mail and/or WhatsApp^®^ message, containing an invitation to participate, the objectives of the research, the ethical implications, the procedures to be performed for data collection and a link to access the survey’s online page.

The message was sent to pregnant women who attended the prenatal care consultation at the outpatient clinic in the previous week. For these, the researchers triggered the message weekly, totaling a maximum of three submissions. After this period, non-response on the survey’s online page was considered as refusal to participate. In total, messages were sent to 1,779 pregnant women, until reaching the number of 151 participants. The scope of the survey was monitored on the survey’s online page, which allowed viewing the responses and the number of participants who responded to data collection.

The online survey page was developed by the researchers on the G Suite® platform using Google Forms®, which stored the electronic form for data collection. On this page, the pregnant woman was directed to read the explanations about the research, the ethical aspects and the Informed Consent Form (ICF). The pregnant woman who agreed to participate in the research expressed her agreement electronically, by clicking on the participation acceptance button on the online page, and automatically received a copy of it by email.

Subsequently, the pregnant woman answered the online electronic form, composed of a participant characteristics instrument and the Depression During Pregnancy Risk Scale (ERDEG)^(12)^, which were self-completed, i.e., the participant read and answered directly without the intervention of the researcher.

The instrument for characterizing the participants was composed of objective questions, authored by the researchers, containing sociodemographic, economic and obstetric variables.

The ERDEG is an instrument to assess the risk of depression in pregnancy, through self-report, developed and validated in Brazil. It consists of 24 questions with dichotomous answers. Each question has two possible answers, with scores ranging from 0 to 1, with the presence of the risk factor corresponding to a score of one, and its absence, to a score of zero. Thus, the total score of the scale ranges from zero to 24 points. The risk of depression in pregnancy identified through the scale is allocated according to the scores obtained from the sum of each question in: lower risk of developing depression in pregnancy (0 – 4 points) and higher risk of developing depression in pregnancy (5 points or more)^(12)^.

### Pilot Test

The electronic form for online data collection was pilot tested, in order to ensure its understanding, being triggered by message via email and/or WhatsApp® for 30 pregnant women undergoing prenatal care at a high-risk pregnancy outpatient clinic, which corresponds to 20% of the established sample, in order to obtain at the end, at least 10%, i.e., 15 pregnant women. Of these, 20 pregnant women answered the form, which were subsequently discarded from the final sample. The result showed that the electronic form was easily understood, without the need for changes in its wording or format for the pregnant population.

### Data Analysis

For analysis, data were exported from Google Forms^®^, by automatic procedures, to a structured spreadsheet in Microsoft Excel^®^; subsequently, they were analyzed in the R program (R Core Team).

Chi-square test was performed for independence, and Fisher’s exact test, for homogeneity of categorical variables. Categorical variables were described in percentage and absolute numbers. Continuous variables were expressed as mean and standard deviation. After the bivariate analysis, the variables were included in the logistic regression model.

To assess the presence of multicollinearity between the parameters of the logistic regression model, the Variance Inflation factor (VIF) was used with values above 5, indicating the presence of variables with collinearity. The selection of relevant variables, after applying the model’s VIF, was carried out through the stepwise procedure, using the Akaike Information criterion. In the final model, the corresponding Odds Ratio was calculated from the parameters obtained with a 95% confidence interval for all variables. The significance level of 5% (α = 0.05) was considered in all statistical tests.

### Ethical Aspects

The research project was sent to the Research Ethics Committee of the *Universidade de São Paulo* at *Escola de Enfermagem de Ribeirão Preto*, in compliance with Resolution 466/12 of the Brazilian National Health Council, approved, according to Opinion 4,474,220, in December 2020.

## RESULTS

The study included 151 pregnant women in high-risk prenatal care. Participant sociodemographic characteristics are described in Table 1.

**Table 1.** Allocation of pregnant women according to sociodemographic characteristics. Ribeirão Preto, São Paulo, 2021.

Variables	N (151)	%
**Age (years)**		
<20	06	4.0
20 – 29	24	15.9
25 – 29	41	27.2
30 – 34	42	27.8
35 – 39	32	21.2
40 – 44	06	4.0
**Marital status**		
Married/with a partner	124	82.1
Single/without a partner	27	17.9
**Education**		
Incomplete elementary school	18	11.9
Complete elementary school	09	6.0
Incomplete high school	21	13.9
Complete high school	69	45.7
Incomplete higher education	18	11.9
Complete higher education	16	10.6
**Religion**		
Evangelicalism	70	46.4
Catholicism	51	33.8
Atheism	18	11.9
Other	12	8.0
**Occupation**		
Paid work	70	46.3
Housewife	63	41.7
Unemployed	16	10.6
Student	02	1.3
**Color**		
White	70	46.4
Brown	62	41.1
Black	17	11.3
Yellow	02	1.3
**Residence**		
Not owned (rented, borrowed, invaded)	78	51.7
Own	73	48.3

Study participants were predominantly young women with a mean age of 29.95 years (SD ± 5.98), a minimum age of 18 and a maximum of 43. Moreover, they had a mean monthly family income of R$1.78 minimum wages (SD ± 1.41), with a minimum of less than one minimum wage and a maximum of nine minimum wages.

Participant obstetric characteristics are described in Table 2. As for the current and previous gestational history, with regard to the current pregnancy, most participants had a mean gestational age of 28.9 (SD ± 8.15) weeks of pregnancy, a minimum of 5 weeks and a maximum of 39 weeks. In addition to this, women with a mean of 2.64 previous pregnancies (SD ±ta 1.48), minimum of one and maximum of ten predominated. The number of previous births ranged from zero to nine, with a mean of 1.36 (SD ± 1.36) and the number of abortions ranged from zero to four, with a mean of 0.42 (SD ± 0.81).

**Table 2.** Obstetric characterization of participants according to gestational trimester, number of pregnancies, births, abortion history. Ribeirão Preto, São Paulo, 2021.

Variables	N (151)	%
**Trimester**		
1^st^ trimester	10	6.6
2^nd^ trimester	34	22.5
3^rd^ trimester	107	70.9
**Number of pregnancies (classification)**		
1 pregnancy (primigravida)	39	25.8
≥ 2 (multiparous)	112	74.2
**Number of births (classification)**		
0 births (nulliparous)	45	29.8
1 birth (primiparous)	43	28.5
≥ 2 births (multiparous)	63	41.7
**Abortion history**		
Yes	41	27.2
No	110	72.8

With regard to the results related to the variable of interest, the measure of risk of depression in pregnancy, obtained through the application of the ERDEG, considered the possible intervals ranging from zero to 24, with the highest value indicating a higher risk of depression in pregnancy. Thus, the interval obtained for the measurement of risk of depression in pregnancy was between zero and 17. The group mean for risk of depression was 7.81 (SD ± 3.59).

Regarding the occurrence of risk of depression during pregnancy, considering the criteria suggested for using the scale^(12)^, which determines the score five as the cut-off point, it was evidenced that pregnant women had a higher risk of depression during pregnancy, with higher rates in the first trimester, as described in Table 3.

**Table 3.** Allocation of pregnant women according to gestational age and risk of depression in pregnancy – Ribeirão Preto, São Paulo, 2021.

Variable	Lower risk of depression N (%)	Higher risk of depression N (%)	p*
**Gestational age**			
1^st^ trimester	26 (78.8)	81 (68.6)	
2^nd^ trimester	2 (6.1)	08 (6.8)	0.494
3^rd^ trimester	5 (15.2)	29 (24.6)	
**Total**	33 (21.9%)	118 (78.1%)	

Note: * = p-value calculated by Pearson’s chi-square test.

Table 4 presents the variables that showed a statistically significant association with the occurrence of a higher risk of depression in pregnancy.

**Table 4.** Factors associated with the risk of depression during pregnancy – Ribeirão Preto, São Paulo, Brazil, 2021.

Variable	Lower risk of depression in pregnancy N (%)	Higher risk of depression in pregnancy N (%)	p	95% OR	CI
**Number of pregnancies**					
1 pregnancy (primigravida)	13 (39.4)	26 (22)	0.047^a^*	0.43	0.19 – 0.99
≥ 2 (multiparous)	20 (60.6)	92 (78)		1.00	
**Marital status**					
Married/with a partner	124	82.1	0.034^a^*	0.11	0.01 – 0.85
Single/without a partner	24	15.9		1.00	
**Residence**					
Not owned	12	66	0.050^a^*	2.22	1 – 4.93
Own	21	52		1.00	

Note: OR = Odds Ratio; CI = Confidence Interval; a = application of Pearson’s chi-square test; *Statistically significant difference for P ≤ 0.05.

The results in Table 4 indicate that pregnant women who were pregnant for the first time (primiparous) had a 57% (1 – 0.43) lower chance of presenting a higher risk of depression during pregnancy than pregnant women who were previously pregnant (multiparous). Similarly, pregnant women who were married/had a partner expressed a 89% (1 – 0.11) lower chance of presenting a higher risk of depression during pregnancy than pregnant women who were single/had no partner. And pregnant women who did not have their own home were 2.22 times more likely to present a higher risk of depression during pregnancy than those who had their own home.

In the final model, as described in Table 5, only the variables number of pregnancies (primiparous) and marital status (married/with partner) remained significantly associated with higher risk of depression in pregnancy, being configured as protective factors.

**Table 5.** Logistic regression model: variables associated with the risk of depression in pregnancy. Ribeirão Preto, São Paulo, Brazil, 2021.

	Estimate	Standard error	p	OR	95% CI
Number of pregnancies (primiparous)	–1.113	0.493	0.024	0.32*	0.12 – 0.85
Marital status (married/with partner)	–2.621	1.064	0.013	0.07*	0.00 – 0.37
Age (≥ 35 years)	0.0954	0.7909	0.903	1.10	0.22 – 5.41
Age (25 to 34 years)	–1.0701	0.5970	0.073	0.34	0.09 – 1.03
Constant	4.599	1.221	0.000		

Note: *Statistically significant difference for P ≤ 0.05.

## DISCUSSION

Pregnant women with obstetric complications, defined as high-risk pregnancies, are at higher risk of developing prenatal depression^(14)^, since prenatal depression has been associated with obstetric parameters^(15)^.

According to a scope review, the prevalence of prenatal depression in high-risk pregnancies ranged from 12.5 to 44.2%, and may differ between studies^(14)^. However, no data were found from previous studies on the risk of developing depression among pregnant women undergoing follow-up in high-risk prenatal care, as assessed in this study. By assessing the risk, not the occurrence of the disorder, through a specific instrument, this study can make an important contribution to the understanding and prevention of the depressive phenomenon during pregnancy.

In a high-risk pregnancy environment, the sociodemographic characteristics of women at risk of depression during pregnancy may differ. Thus, participant characteristics in this study are similar to that of other studies developed in the context of high-risk prenatal care regarding age, marital status and occupation^(16)^. However, it differs from another study regarding income^(17)^.

Among the participating pregnant women, 78.1% had a higher risk of developing depression during pregnancy. When considering the risk of depression, data are scarce, since the other studies assessed the prevalence of depressive disorder and/or its symptoms, and not the risk of its occurrence among pregnant women. Nevertheless, the findings of this study are in line with other studies, since the higher risk of depression in pregnancy among high-risk pregnant women corroborates the higher prevalence of the disorder in this environment. In this sense, a previous scoping review reported rates of major antenatal depression among pregnant women with high-risk pregnancies ranging from 12.5 to 44.2%^(14)^.

It should be noted that methodological differences between studies and different measurement tools may attribute differences in rates of depression between studies, and sociodemographic aspects and economic differences may also be responsible for the difference in prenatal depression rates between results from different countries.

In this regard, It is important to emphasize that the high rate of higher risk of depression in pregnancy found in the present study can be explained by the fact that the Risk of Depression in Pregnancy Scale is a self-completed instrument that was applied online and, as a result, the women probably did not feel influenced by the researcher and more easily expressed what they really felt about the risk of depression.

Furthermore, data were collected during the COVID-19 pandemic, during which time pregnant women could present additional anxiety and depression, and consequently, increase their risk. This fact corroborates a previous study that demonstrated an increase in depressive symptoms and psychological distress in women with high-risk pregnancies during the COVID-19^(18)^ pandemic.

In the present study, gestational age was not significantly associated with the risk of depression during pregnancy, but this was more frequent in the first trimester of pregnancy. This result is similar to the data evidenced in a cohort of 1,000 days (S1000) carried out in Soweto, South Africa^(19)^ and contrasts with a study conducted in Indonesia, where depression was more frequent among pregnant women in the third trimester^(20)^.

In view of this finding, the importance of early intervention in the first trimester of pregnancy is highlighted, which can have secondary preventive effects, in addition to reducing costs for the health system.

The number of pregnancies (primiparous) and marital status (married/with partner) were factors that protected the risk of depression during pregnancy. However, in a study conducted with pregnant women hospitalized in a high-risk pregnancy unit in Greece, these associations were not verified^(21)^.

It should be noted that primigravidae experiences pregnancy for the first time and, although it is associated with pre-existing complications of a high-risk context, this does not occur under the influence of negative experiences from previous pregnancies that could contribute to developing and/or increasing the risk of depression.

On the other hand, the presence of a partner provides social support to women during pregnancy and brings more power to deal with pregnancy and domestic responsibilities. In contrast, women with unfavorable marital conditions, such as being single or having gone through a divorce process, may live alone, experience more loneliness and low self-confidence, which can predispose to depression^(22)^.

Living in a non-own house was also associated with a higher risk of depression during pregnancy, which demonstrates the impact of social determinants on women’s mental health, especially in environments marked by socioeconomic and life inequalities^(23)^.

In the multivariate analysis, it was evidenced that being primiparous and having a partner or being married are protective factors against a higher risk of depression during pregnancy. In this context, it is worth mentioning the complexity of the phenomenon under study, the importance of considering the effect of the variables together on the higher risk of depression in pregnancy and the sociocultural setting of society in which the pregnant women studied are inserted.

Similar data was reported in an African study, in which the presence of a partner who provides social support and is seen as a confidant to listen and talk to the pregnant woman significantly reduced the chances of depression^(19)^.

The study limitation is its cross-sectional design, which makes inferences about the temporal relationship of events or the cause-and-effect relationship of the results found impossible. The study contributes to outlining the vulnerability scenario of pregnant women to depression in pregnancy, helping health professionals involved in prenatal care, especially nurses, who work in primary care settings, constructing prevention and management strategies.

## CONCLUSION

In the stratification proposed by the instrument used, which defines the options “lower risk of depression during pregnancy” and “higher risk of depression during pregnancy”, most pregnant women in this study presented “higher risk of depression during pregnancy”.

In the context of high-risk prenatal care, marital status (married/with a partner) and number of pregnancies (primigravidae) constituted protective factors for a higher risk of depression during pregnancy.

Considering the serious consequences of depression in pregnancy, the results of this study elucidate the importance of screening for the risk of depression and the significant need to improve access to effective interventions to prevent prenatal depression and promote their mental health in pregnant women undergoing high-risk prenatal care.

Identifying pregnant women at risk of depression supports referral to specialized mental health care, when necessary, making it opportune to develop targeted approaches to screening and treatment.

## ASSOCIATE EDITOR

Thiago da Silva Domingos
